# Preventive physiotherapy interventions for back care in children and adolescents: a meta-analysis

**DOI:** 10.1186/1471-2474-13-152

**Published:** 2012-08-21

**Authors:** Inmaculada Calvo-Muñoz, Antonia Gómez-Conesa, Julio Sánchez-Meca

**Affiliations:** 1 Dept. of Physiotherapy, University of Murcia, Murcia, Spain; 2 Dept. of Physiotherapy, University of Murcia, Murcia, Spain; 3 Dept. of Basic Psychology and Methodology, University of Murcia, 30100, Murcia, Spain

**Keywords:** Physiotherapy, Prevention, Efficacy, Children, Adolescents, Meta-analysis, Back care

## Abstract

**Background:**

Preventive interventions improve healthy behaviours and they also increase knowledge regarding back care in children and adolescents, but studies exhibit great variability in their contents, duration and number of sessions, and in the assessment methods. The purpose of this study was to review the empirical evidence regarding preventive physiotherapy interventions for back care in children and adolescents, and to ascertain the most efficacious treatments, in what way and under which circumstances.

**Methods:**

Studies were located from computerized databases (Cochrane Library, Medline, PEDro, Web of Science and IME) and other sources. The search period extended to May 2012. To be included in the meta-analysis, studies had to use physical therapy methodologies of preventive treatment on children and adolescents, and to compare a treatment and a control group. Treatment, participant, methodological, and extrinsic characteristics of the studies were coded. Two researchers independently coded all of the studies. As effect size indices, standardized mean differences were calculated for measures of behaviours and knowledge, both in the posttest and in the follow-up. The random and mixed-effects models were used for the statistical analyses and sensitivity analyses were carried out in order to check the robustness of the meta-analytic results.

**Results:**

A total of 19 papers fulfilled the selection criteria, producing 23 independent studies. On average, the treatments reached a statistically significant effectiveness in the behaviours acquired, both in the posttest and in the follow-up (*d*_+_ = 1.33 and *d*_+_ = 1.80, respectively), as well as in measures of knowledge (posttest; *d*_+_ = 1.29; follow-up: *d*_+_ = 0.76). Depending on the outcome measure, the effect sizes were affected by different moderator variables, such as the type of treatment, the type of postural hygiene, the teaching method, or the use of paraprofessionals as cotherapists.

**Conclusions:**

The interventions were successful in significantly increasing the behaviours and knowledge acquired both in the posttest and in the follow-up. The combined treatment of postural hygiene with physiotherapy exercise exhibited the best results. The small number of studies limits the generalizability of the results.

## Background

Epidemiological studies indicate that non-specific low back pain (LBP) is already present during childhood [1] and it is one of the main reasons for suffering chronic LBP as an adult [2]. Many studies have shown a great prevalence of LBP in children and adolescents [3-7]. According to the literature on the epidemiology of LBP in children and adolescents, estimates of the lifetime prevalence vary between 8.6% [8] and 58.90% [6].

The risk of developing LBP depends on several factors [9]. Lifestyle-related factors, anthropometric factors, school-related factors and psychosocial factors are all associated with LBP in children and adolescents [2,10,11]. Programs for the prevention of LBP and discomfort have mainly been carried out on the adult population [12-15], fundamentally due to the associated expenses that this disorder generates. In recent decades, as a result of the increase in morbidity of back problems in children and adolescents, a need has been detected to develop preventive interventions for this population group [16]. In answer to this need, the European Region of the World Confederation for Physical Therapy (ER-WCPT) has recently published the results of a study carried out in the European Union [17]. There is evidence that the preventive approach produces an increase in the acquisition of knowledge and an improvement in appropriate postural habits that favour back care in children and adolescents [18-21].

Preventive interventions for children and adolescents have been aimed at increasing the cognitions related to protecting the back in everyday activities (at school, at home, and in sports) through different methods of teaching and learning [22,23]. The preventive interventions that have been employed include physical therapy exercises to improve physical fitness [19,20,24], training in positions and movements used in everyday activities for a healthier back (to avoid overloading) [18,25-29] and, more recently, increasing physical activity [17,30].

The school has been the location for numerous research studies on the development of back care interventions in this population [22,25,26,31,32], although interventions have varied considerably in many aspects, such as the type of intervention, teaching techniques, duration, magnitude and intensity of sessions, mode of intervention, characteristics of the participants, and how the interventions are assessed.

Due to the lack of previous meta-analyses in this context, our main objective was to assess the evidence regarding preventive physiotherapy interventions for back care in children and adolescents, and to ascertain which ones prove to be the most efficacious, in what way and under which circumstances. Our specific objectives were: (a) to estimate the efficacy of preventive physiotherapy interventions for back care in children and adolescents, and (b) to examine the influence of treatment, participant, methodological, contextual, and extrinsic characteristics of the studies on the effect size.

Starting from the literature on this subject, several hypotheses were formulated: (a) the intensity, magnitude and duration of treatment will be positively related to the results; (b) treatments that include external agents will obtain better results; (c) treatments that include the parents or teachers will attain greater effect sizes; (d) the sex of participants will influence results, in that girls will acquire greater knowledge than boys [33,34]; (e) the age of participants will influence the effect sizes, greater knowledge being expected amongst older children and knowledge being improved when behaviours are acquired from a younger age, and (f) the type of control group has an influence on the effect size, as studies with a nonactive control group will produce higher effect sizes than studies with an active control group.

## Methods

### Study selection

The studies had to fulfil the following criteria to be selected: (a) the study had to apply some physical therapy methodology of preventive treatment for LBP; (b) the participants in the study had to pertain to a nonclinical population of children and/or adolescents aged below 19 years; (c) the study had to include, at least, a treatment and a control group; (d) the minimum sample size in the posttest had to be of 5 subjects per group; (e) the study had to report enough statistical data to calculate the effect sizes; (f) the study had to be published or carried out before May 2012; (g) the study might be written in English, Spanish, French, Italian, Portuguese, and Catalan. Finally, studies in which all subjects in the sample presented pain, spinal diseases or surgical vertebral treatment were excluded, since our focus was on preventive interventions.

### Data sources and searches

Combined search processes were used for locating the studies, clearly planned and ordered. The following specialized bibliographical databases were consulted: the Cochrane Library, Medline, PEDro, Web of Science and IME (Spanish Medical Index). The search period extended to May 2012, with the following key words: children, adolescents, treatment, prevention, education, “postural hygiene”, “physical education”, “back education”, “posture education”, “back function”, physiotherapy, ergonomics, ”physical therapy”, “exercise therapy”, promotion, behaviour, “back care”, “back pain”, “low back pain”. For details regarding the search terms and combinations, see Additional file 1. Journals from the Elsevier Iberoamerican database were also consulted, as well as specialized electronic journals. References of relevant papers already identified were consulted and, in order to locate unpublished studies, letters were sent to experts in the field and congress acts and doctoral theses were consulted.

A total of 956 references were located, from which 905 were excluded in a first screening. The main reasons for deleting these studies were because the participants in the samples were adults (about 50%) or pertained to clinical populations, such as diseases that cause back pain (about 15%), because of applying pharmacological treatments for LBP (about 20%), or by other reasons (about 15%). The reading of the remaining 62 papers allowed us to identify 19 articles that fulfilled the selection criteria. The Additional file 2 presents the flow chart of the selection process of the studies. Given that some papers included two groups that were receiving alternative treatments and a control group, a total of 23 studies were included, with a study being defined as a comparison between a treatment and a control group.

### Data extraction and quality assessment

In order to assure the maximum possible objectivity, a codebook was produced that specified the standards followed in coding each of the characteristics of the studies. The moderator variables of the 23 studies were coded and grouped into three categories according to Lipsey’s recommendations [35]: substantive (treatment, context and participant), methodological, and extrinsic variables.

The following treatment characteristics were coded: (a) the type of preventive physiotherapy treatment (postural hygiene, physiotherapy exercise, physical activity); (b) the acquisition mode of postural hygiene (acquisition of knowledge, posture training habits); (c) the teaching method of postural hygiene (theoretical, practical); (d) the type of physiotherapy exercise (stretching, strengthening, pelvic tilt exercises, breathing, posture correction, balance exercises); (e) the type of physical activity (sports, games); (f) the duration of the treatment (in weeks); (g) the intensity of the treatment (number of weekly hours of treatment received by each subject); (h) the magnitude of the treatment (total number of hours received by each subject); (i) the existence of an established number of sessions; (j) the homogeneity of the treatment (whether all patients received the treatment in the same conditions; (k) the inclusion of homework; (l) the inclusion of a follow-up program; (m) the use of external agents to the therapeutic group (subjects that are not part of the therapy group, who are not professionals, but who have an influence, being able to support the subjects in attaining their therapy goals); (n) the presence of family members who act as cotherapists that continue or carry out preventive treatment at home); (o) the presence of teachers who act as cotherapists that continue or carry out preventive treatment at home; (p) the mode of application of the intervention (direct, indirect or mixed); (q) the mode of training (group, individual or mixed); (r) the use of informed consent. Regarding the characteristics of the therapists the following variables were coded: (s) the number of therapists; (t) whether or not the authors agree with the therapists; (u) the training of the therapist (physiotherapist, other); (v) the experience of the therapists (large, medium, low, mixed), and (w) the gender of therapists (men, women, mixed).

The participant characteristics coded in the samples of each study were: (a) the mean age of the subjects (in years); (b) the gender of the sample (percentage of males); (c) the physical activity level of subjects during the intervention (low, moderate, regular), and (d) whether or not they had undertaken previous treatments. Only two contextual characteristics were coded: (a) the country and (b) the place where the intervention was carried out (university, clinic, health centre / day centre, hospital, school, sports centre, mixed).

The following methodological characteristics were coded: (a) whether pretest measures were used; (b) how the subjects were allocated to the treatments (randomly vs. nonrandomly); (c) the type of control group (nonactive vs. active); (d) the largest follow-up in the study (in months); (e) the sample size; (f) the attrition in the posttest; (g) the attrition in the follow-up; (h) the methodological quality of the study measured on a scale of 0 to 8 points following van Tulder [36] but with a few adaptations to our selected studies (the scale consisted of adding the scores of eight items: random assignment, control group type, sample size, attrition, intent-to-treat analysis, evaluator blinding, homogeneous assessment, and inter-rater reliability).

Finally, the extrinsic characteristics coded were: (a) the year of the study; (b) the profession of the first author (physiotherapist, ergonomist, teacher, physician, other) and (c) the publication source (published vs. unpublished). In addition, given that the studies included in the meta-analysis came from a few research teams, this characteristic was also coded in order to examine its potential influence on the study results.

In order to assess the inter-coder reliability of the coding process, two researchers (A.G.C. and I.C.M.) independently coded all of the studies. For the quantitative moderator variables intra-class correlation coefficients were calculated (ICC), while for the qualitative moderator variables Cohen’s kappa coefficients were applied. On average, the ICC was 0.995 (range: 0.954 to 1) and the kappa coefficient was 1, which were highly satisfactory, as proposed by Orwin and Vevea [37]. The inconsistencies between the coders were solved by consensus and the coding manual was corrected when the cause of these inconsistencies was due to an error in it. The codebook can be obtained from the corresponding author.

### Effect size index

The standardized mean difference, *d*[38], was used as the effect size index, adhering to the following definitions according to whether or not the study included pretest measurements: when the study did not include pretest measurements, a standardized mean difference was calculated, defined as the difference between the treatment and control means in the posttest, divided by a pooled within-group standard deviation. The same index was applied for the follow-up measurements. When the study included pretest measurements, the effect size index was the standardized mean change [39], defined as the difference between the pretest-posttest mean change for the treatment and control groups, divided by a pooled estimate of the pretest standard deviations of the two groups. Similar effect sizes were calculated from the pretest-follow-up measurements. Four studies compared two alternative treatments with the same control group, so that the data from the control group were used twice in the effect size calculations [22,27,40,41]. In order to minimize the dependence produced by sharing the control group [42], its sample size was divided in two.

Separate effect sizes were calculated for two different outcomes: behaviours and knowledge measurements. Thus, from each study four effect sizes might be calculated: behaviours and knowledge in the posttest, and behaviours and knowledge in the follow-up. The effect sizes were calculated from means, standard deviations and other statistics, such as *T*-tests, *F*-tests, etc. [43,44]. In order to check the reliability of the effect size calculations, two independent researchers (J.S.M and I.C.M) carried out the calculations for all of the studies, reaching an average intra-class correlation coefficient of 0.950 (range: 0.701-1), which were also highly satisfactory [37].

### Data analysis

With the effect sizes obtained for behaviours and knowledge, both in the posttest and in the follow-up, separate meta-analyses were carried out. This implied to construct a forest plot, to obtain a mean effect size with its 95% confidence interval, and to assess the effect sizes’ heterogeneity with the *Q* statistic and the *I*^2^ index [45]. For these calculations, a random-effects model was applied and this implied to weight each effect size by its inverse-variance, with the variance defined as the sum of the within-study and the between-studies variances [38]. Sensitivity analyses were carried out in order to assess the robustness of the meta-analytic results. Thus, the influence of outlying effect sizes was assessed by deleting them from the statistical analyses, in order to check whether a few data might affect the results. In addition, funnel plots were constructed and the trim-and-fill method [46] was applied, to assess whether publication bias might be a threat to the validity of the meta-analytic results. When the meta-analysis included, at least, 10 studies, the influence of moderator variables was checked by applying mixed-effects analyses of variance (ANOVAs), for the qualitative variables, and simple meta-regressions, for the quantitative ones. In the ANOVAs and meta-regressions, *Q*_B_ and *Q*_R_ statistics were calculated, respectively, to assess the statistical significance of the moderator variables, and *Q*_W_ and *Q*_E_ statistics to assess the model misspecification. To estimate the effect magnitude of each moderator variable on the effect sizes, the proportion of variance accounted for proposed by Raudenbush [47] was applied: $R^{2}=1-{\hat{\tau }}_{\mathrm{Res}}^{2}/{\hat{\tau }}_{\mathrm{Total}}^{2}$ , with ${\hat{\tau }}_{\mathrm{Total}}^{2}$ and ${\hat{\tau }}_{\mathrm{Res}}^{2}$ being the total and residual between-studies variances, respectively [38]. The statistical analyses were made using the meta-analysis macros developed by David B. Wilson for the statistical package SPSS [48]. The forest plots were carried out with RevMan 5.1 [49], and the funnel plots with the trim-and-fill method were obtained from the package *Comprehensive Meta-analysis 2.0*[50]. The PRISMA checklist [51] was used to check the reporting quality of the meta-analysis (Additional file 3).

## Results

### Descriptive characteristics of the studies

Nineteen papers fulfilled the selection criteria [19-24,27-30,33,34,40,41,52-56], generating a total of 23 independent studies each one comparing a treatment and a control group. The studies were carried out between 1984 and 2011, two of them being unpublished papers (Additional file 2). Eleven studies were carried out in Belgium, seven in Spain, two in USA and South Korea and one in Brazil. In the pretest, a total of 4,519 participants were distributed into 21 treatment groups (2,554 participants; median: 44) and 16 control groups (1,965 participants; median: 50). In the posttest, the total sample size was of 4,423 participants, 2,493 pertaining to the treatment groups (median: 42) and 1,930 to the control groups (median: 50). In the follow-up, the total sample size reduced to 2,605 participants, distributed into 12 treatment groups (1,344 participants; median: 84) and 11 control groups (1,261 participants; median: 98).

The individual characteristics of each of the integrated studies are presented in Table 1. In relation to the type of intervention, the most noteworthy was postural hygiene applied on its own (19 studies), in comparison with the combined treatment of postural hygiene and physical therapy exercises (three studies) and postural hygiene and physical activity (one study). The median number of weeks of intervention was 6, the median intensity was one hour per week and the median magnitude was 4.5 hours. The mean age of participants in the samples was 11.3 years and the mean percentage of males was 48.1%. Out of the 23 studies, 20 of them included pretest measurements. With regards to the methodological quality of the studies, the mean score obtained with the quality scale (range: 0–8) was 6.1 (minimum: 3.4, maximum: 7.5). The results of the critical appraisal for the selected studies are presented in Additional File 4. As all of the studies were carried out in schools, it was not possible to randomly assign the subjects to the experimental conditions, but in all of them the decision regarding which group received the intervention or the control condition was at random, with the exception of one study [41]. Only in three studies [20,23,40] was an active control group used, with the remaining studies using a nonactive one. In eight studies [21,29,30,52-56] there was attrition in the experimental group and all of them reported intent-to-treat analyses, with the exception of one study [56]. In 15 studies [19-21,23,27-30,33,34,40,52-55] the assessor was blinded. All of the studies assessed the subjects in the same conditions (e.g., at the same time), and four studies [22,24,40,56] did not report the reliability of the measurement instruments used. Only two studies were unpublished papers [33,34]. The most frequent profession for the first author was physiotherapist (18 studies) and the studies were carried out between 1984 and 2011.

**Table 1:** Characteristics of the studies included in the meta-analysis

**Papers (19)**	**Studies (23)**	**Participants**	**Contents**	**Interventions**	**Outcomes**
Cardon et al., [19] (2000) Belgium		78 subjects Age: 10.2 E = 42 C = 36	German Back School Correct realization of different activities of daily life Stretching and strengthening exercises	E: 6 sessions (60 mins) various tasks based on good understanding of basic back care principles (guided discovery and hands-on methods) + 2 hours with the participation of their parents and teachers	The experimental group obtained higher scores than the control group for the behaviors and knowledge in the posttest
				C: control	
Cardon et al., [27] (2001) Belgium	(a)	72 subjects 5th grade Age: 11 E = 38 C = 34	German Back School Correct realization of different activities of daily life Stretching and strengthening exercises	E: 6 sessions (60 mins; once a week) various tasks based on good understanding of basic back care principles (guided discovery and hands-on methods) + extra guidelines of their teachers to integrate the learned principles (12 weeks)	The experimental group obtained higher scores than the control group for the behaviors and knowledge
				C: control	
	(b)	82 subjects 5th grade Age: 11 E = 48 C = 34	German Back School Correct realization of different activities of daily life Stretching and strengthening exercises	E: 6 sessions (60 mins; once a week) various tasks based on good understanding of basic back care principles (guided discovery and hands-on methods) without extra guidelines of their teachers	The experimental group obtained higher scores than the control group for the behaviors and knowledge
				C: control	
Cardon et al., [28] (2002a) Belgium		706 subjects Age: 10.02 (9–11) E = 347 C = 359	German Back School Correct realization of different activities of daily life Stretching and strengthening exercises	E: 6 sessions (60 mins; once a week) various tasks based on good understanding of basic back care principles (guided discovery and hands-on methods) + 2 hours with the participation of their parents and teachers	Intervention children showed better back care knowledge than control children, and knowledge gained was retained over a period of one year
				C: control	
Cardon et al., [29] (2002b) Belgium		363 subjects 4th, 5th grade Age: 9–12 E = 198 C = 165	German Back School Correct realization of different activities of daily life Stretching and strengthening exercises	E: 6 sessions (60 mins; once a week) various tasks based on good understanding of basic back care principles (guided discovery and hands-on methods) + 2 hours with the participation of their parents and teachers	The experimental group obtained higher scores than the control group for the behaviors
				C: control	
Cardon et al., [30] (2007) Belgium		362 subjects 4th, 5th grade E = 190 C = 172	German Back School. Correct realization of different activities of daily life Stretching and strengthening exercises Physical activity: sports, play and active recreation for kids (SPARK)	E: 6 sessions (60 mins; once a week) various tasks based on good understanding of basic back care principles (guided discovery and hands-on methods) + extra guidelines from their teachers to integrate the principles learned and to increase postural dynamics (2 school years) + physical activity promotion program	The experimental group obtained higher scores than the control group for the behaviors and knowledge
				C: control	
Cardoso [33] (2009) Brazil		519 subjects Age: 8–21 E = 269 C = 250	Spinal care principles and how to incorporate this knowledge into everyday life Behavioral intervention	E: 4 sessions (twice week) Lecture, demonstration, hands on practice (2 weeks)	The experimental group obtained higher scores than the control group for the knowledge
				C: control	
Dolphens et al., [52] (2011) Belgium		194 subjects Age: 18 E = 96 C = 98	German Back School Correct realization of different activities of daily life Stretching and strengthening exercises	E: 6 sessions (60 mins; once a week) various tasks based on good understanding of basic back care principles (guided discovery and hands-on methods) + 2 hours with the participation of their parents and teachers	The experimental group obtained higher scores than the control group for the knowledge
				C: control	
Geldhof et al., [21] (2006) Belgium		365 subjects 4th, 5th grade E = 193 C = 172	German Back School Correct realization of different activities of daily life Stretching and strengthening exercises	E: 6 sessions (60 mins; once a week) various tasks based on good understanding of basic back care principles (guided discovery and hands-on methods) + extra guidelines of their teachers to integrate the learned principles and to increase postural dynamics (2 school years)	The experimental group obtained higher scores than the control group for the behaviors and knowledge
				C: control	
Geldhof et al., [53] (2007a) Belgium		69 subjects Age: 8–11 E = 41 C = 28	German Back School Correct realization of different activities of daily life Stretching and strengthening exercises	E: 6 sessions (60 mins; once a week) various tasks based on good understanding of basic back care principles (guided discovery and hands-on methods) + extra guidelines of their teachers to integrate the learned principles and to increase postural dynamics (2 school years)	The effects of 2 years back education showed an increase in trunk flexor endurance in the experimental group compared to a decrease in the controls
				C: control	
Geldhof et al., [54] (2007b) Belgium		195 subjects 7th, 8th grade E = 94 C = 101	German Back School Correct realization of different activities of daily life Stretching and strengthening exercises	E: 6 sessions (60 mins; once a week) various tasks based on good understanding of basic back care principles (guided discovery and hands-on methods) + extra guidelines of their teachers to integrate the principles learned and to increase postural dynamics (2 school years)	The experimental group obtained higher scores than the control group for the knowledge in the follow-up (2 years)
				C: control	
Geldhof et al., [55] (2007c) Belgium		245 subjects 6th,7th grade E = 121 C = 124	German Back School Correct realization of different activities of daily life Stretching and strengthening exercises	E: 6 sessions (60 mins; once a week) various tasks based on good understanding of basic back care principles (guided discovery and hands-on methods) + extra guidelines of their teachers to integrate the learned principles and to increase postural dynamics (2 school years)	The experimental group obtained higher scores than the control group for the knowledge in the follow-up (1 years)
				C: control	
Gómez and Méndez [23] (2000a) Spain		67 subjects 5th grade Age: 11 E = 33 C = 34	Anatomy, biomechanics, respiratory mechanism and the way to avoid column overload, risk factors for injury, spinal care principles	E: 8 sessions (30 mins; once a week). Lecture to incorporate knowledge about the correct functioning of the body and to avoid vertebral overload and back injuries from childhood.	The experimental group obtained higher scores than the control group for the knowledge in the posttest and the follow up carried out in 6 months time.
				C: performed with their academic tutor-teacher about related matters.	
Gómez and Méndez [40] (2000b) Spain	(a)	65 subjects 5th grade Age: 11 E = 33 C = 32	Postural hygiene, how to incorporate this knowledge into everyday life Behavioral intervention	E: Information and training from a physiotherapist + parents were given information about postural hygiene, training in observation and healthy motive habits registration (2 hours)	The experimental group obtained higher scores than the control group for the behaviors
				C: parents were given information about postural hygiene, training in observation and healthy motive habits registration (2 hours)	
	(b)	66 subjects 5th grade Age: 11 E = 34 C = 32	Postural hygiene, how to incorporate this knowledge into everyday lifeBehavioral intervention	E: Piece of ergonomics advice from a tutor + parents were given information about postural hygiene, training in observation and healthy motive habits registration (2 hours)	The experimental group obtained higher scores than the control group for the behaviors
				C: parents were given information about postural hygiene, training in observation and healthy motive habits registration (2 hours)	
Kovacs et al., [56] (2011) Spain		574 subjects Age: 8 E = 320 C = 254	Comic Book of the Back	E: Comic Book of the Back handed over by teachers	The experimental group obtained higher scores than the control group for the knowledge
				C: control	
Martínez [34] (2007) Spain		579 subjects (3rd- 6th grade) Age: 7–12 E = 314 C = 265	Spinal care principles and how to incorporate this knowledge into everyday life Behavioral intervention	E: 5 sessions (once week) Lecture, demonstration, hands on practice (5 weeks)	The experimental group obtained higher scores than the control group for the behaviors and knowledge in the posttest
				C: control	
Méndez and Gómez [20] (2001) Spain		70 subjects 3rd grade Age: 9 E = 35 C = 35	Anatomy, biomechanics, risk factors for injury, spinal care principles Behavioral intervention Correct realization of different activities of daily life Stretching and strengthening exercises	E: 11 sessions (60 mins; once a week): 8 sessions (postural hygiene knowledge and behaviors; each lasted 2 hours) and 3 sessions (physiotherapy exercise; each lasted 1 hour), total of 19 hours	The experimental group obtained higher scores than the control group for the knowledge and behaviors in the posttest and the follow up carried out after 12 months
				C: took part in different academic activities with related themes	
Park and Kim [41] (2011) South Korea	(a)	59 subjects 5th grade Age: 11 E = 28 C = 31	Web-based spinal health education program (anatomy, functions of the spine, spinal care principles, stretching and strengthening exercises, backpack use)	E: 4 sessions (30 mins; once a week) Web-based program 3 parts (learning, formative evaluation, learning summary sections) (4 weeks)	The changes for spinal health knowledge were significantly higher than those of the control group.
				C: control	
	(b)	60 subjects 5th grade Age: 11 E = 29 C = 31	Face-to-face spinal health education program (anatomy, functions of the spine, spinal care principles, stretching and strengthening exercises, backpack use)	E: 4 sessions (30 mins; once a week) Face-to-face instruction 3 parts (learning, formative evaluation, learning summary sections) (4 weeks)	The changes for spinal health knowledge were significantly higher than those of the control group.
				C: control	
					
					
					
Spence et al., [22] (1984) US	(a)	50 subjects 3rd, 5th grade E = 25 C = 25	Safe lifting techniques	E: 1 session: lecture demonstration (5 mins videotape), review of the major principles presented in the tape (5 mins)	Showed significantly higher knowledge in the experimental group versus the control group in the posttest, but no significant differences between groups in the follow-up (2 months) Behaviours: Provided inconclusive or statistically insignificant results
				C: without intervention	
	(b)	51 subjects 3rd, 5th grade E = 26 C = 25	Safe lifting techniques	E: 1 session: guided self discovery (15 mins)	Showed significantly higher knowledge in the experimental group versus the control group in the posttest, but no significant differences between groups in the follow-up (2 months) Behaviours: Provided inconclusive or statistically insignificant results
				C: without intervention	
Vidal et al., [24] (2009) Spain		137 subjects Age: 10–12 E = 63 C = 74	Anatomy, biomechanics, risk factors for injury, spinal care principles, respiratory mechanism, postural hygiene Behavioral intervention Exercise	E: 6 sessions: 4 sessions (knowledges: anatomy, biomechanics, risk factors for injury, spinal care principles, respiratory mechanism, postural hygiene) and 2 sessions (behavioral intervention, exercise); total of 4 weeks	The experimental group obtained higher scores than the control group for the knowledge
				C: without intervention	

E: experimental group / C control group.

All studies are randomized controlled.

### Mean effect size and heterogeneity analysis

**Figure 1:**
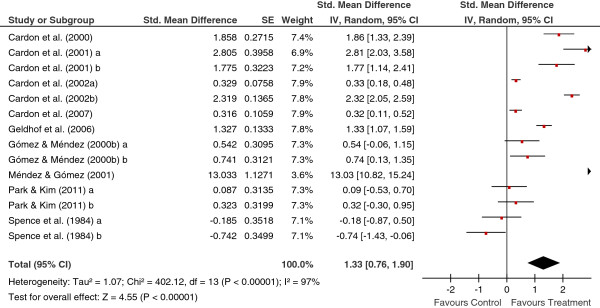
Forest plot of effect sizes for measures of behaviours in the posttest.

**Figure 2:**
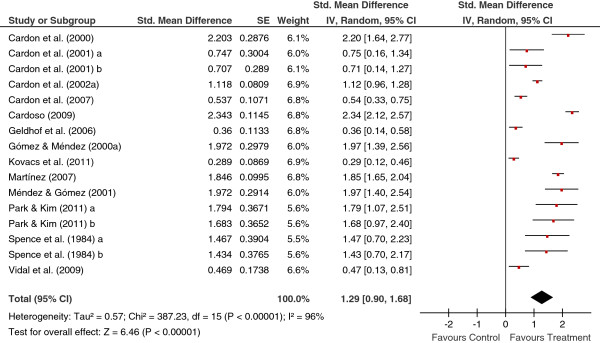
Forest plot of effect sizes for measures of knowledge in the posttest.

**Figure 3:**
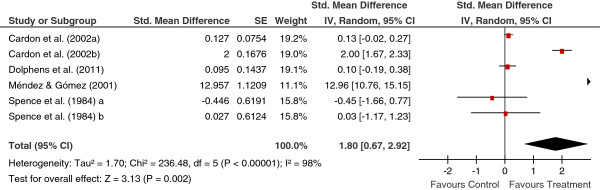
Forest plot of effect sizes for measures of behaviours in the follow-up.

**Figure 4:**
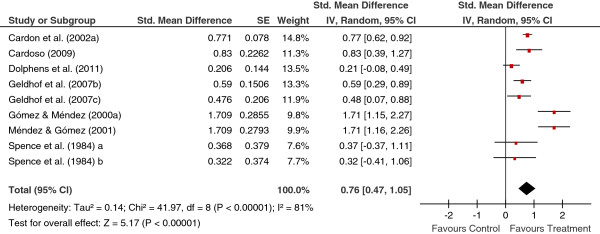
Forest plot of effect sizes for measures of knowledge in the follow-up.

The main measure of treatment effectiveness was the effect size obtained in the posttest and in the follow-up for the outcome measures of behaviours and knowledge. Separate meta-analyses were carried out for each combination of outcome measure and time point. Table 2 shows the main results for the four meta-analyses, and Figures 1, 2, 3, 4 present a forest plot for each one of them. Overall, the four average effect sizes were positive in favour of the treatments (Table 2). Furthermore, all of the mean effect sizes were of a large magnitude according to the Cohen’s criteria [57], as they were over or close to 0.8.

**Table 2:** Mean effect size and heterogeneity analysis for the two outcome measures in the posttest and follow-up.

**Time point / Outcome measure**	***k***	***d***_**+**_	**95% C. I**		***Q***	***df***	***p***	***I***^**2**^
			***d***_**l**_	***d***_**u**_				
Posttest: Behaviours	14	1.328	0.756	1.901	402.12	13	< .001	97%
Posttest: Knowledge	16	1.288	0.898	1.679	387.23	15	< .001	96%
Follow-up: Behaviours	6	1.795	0.672	2.919	236.48	5	< .001	98%
Follow-up: Knowledge	9	0.762	0.473	1.050	41.97	8	< .001	81%

*k*: number of studies. *d*_+_: mean effect size. *d*_l_ and *d*_u_: lower and upper confidence limits of the 95% confidence interval around the mean effect size. *Q*: heterogeneity statistic. *df*: degrees of freedom. *I*^2^: heterogeneity index.

Figure 1 presents a forest plot for the behaviour measures in the posttest, with a mean effect size of *d*_+_ = 1.33 (95% CI: 0.76 and 1.90), statistically significant and with the effect sizes exhibiting a large variability (*I*^2^ = 97%). As Figure 1 shows, one of the studies exhibited an outlying effect size of *d* = 13.033 [20]. The reasons for this so different result in comparison to the remaining studies included in the meta-analysis can be found in the characteristics of the intervention implemented. Thus, out of the 23 studies, this one was who exhibited the longest intensity (2.4 hours per week), the largest magnitude (a total of 19 hours of intervention), the only one that included homework; in addition, this study was one of the three that used family cotherapists, and one of the eight that used teachers as cotherapists. A more representative estimate of the treatment effectiveness for the set of studies included in this meta-analysis was obtained by removing this study from the analysis. When this study was removed, the mean effect size decreased to *d*_+_ = 0.89 (95% CI: 0.39 and 1.38), although still being statistically significant and remaining a large heterogeneity (*I*^2^ = 96%). Thus, the inclusion of this study in the analyses implied an increase of 49.4% for the mean effect size (from 0.89 to 1.33). The extremely atypical effect size obtained in this study advises, therefore, to remove it from the moderator analyses.

Figure 2 presents a forest plot for knowledge measures in the posttest, with a mean effect size of *d*_+_ = 1.29 (95% CI: 0.90 and 1.68), statistically significant and with a large magnitude. The 16 studies also showed a large variability (*I*^2^ = 96%).

Twelve studies enabled us to calculate effect sizes from the follow-ups, being the range between two and 96 months, and with a mean of 16.2 months and a median of 11 months. Out of these, six studies reported measures of behaviours. Figure 3 presents the forest plot, with a mean effect size of *d*_+_ = 1.80 (95% CI: 0.67 and 2.92), statistically significant and even larger than that obtained in the posttest. However, the Méndez and Gómez’s (2001) study [20] showed a very outlying effect size of *d* = 12.957. By deleting this study from the analysis, the mean effect decreased to *d*_+_ = 0.44 and it did not reach the statistical significance (95% CI: -0.41 and 1.28). This estimate of the true effect of the interventions seems to be more representative of the set of studies included in the meta-analysis.

Nine studies assessed knowledge in the follow-up. Figure 4 presents the forest plot, with a mean effect size of *d*_+_ = 0.76 (95% CI: 0.47 and 1.05), statistically significant and exhibiting a large heterogeneity (*I*^2^ = 81%). Two studies [20,23] exhibited an effect size of *d* = 1.709, slightly over those obtained in the remaining studies. When these two effect sizes were removed from the analysis, the mean effect size decreased to *d*_+_ = 0.55, although maintaining the statistical significance (95% CI: 0.34 and 0.76) and still exhibiting a large heterogeneity (*I*^2^ = 58%).

### Analyzing publication bias

Due to the low representativeness of the unpublished studies in our meta-analysis, we checked whether publication bias could be a threat to the validity of our results by applying an ANOVA to compare the mean effect size of the published vs. unpublished studies for the outcome variable of knowledge in the posttest and in the follow-up. In the posttest, the ANOVA reached a statistically significant difference between the mean effect sizes for the published and unpublished studies [*Q*_B_(1) = 5.86, *p* = .015; *R*^2^ = 0.541], but contrary to the expectations: a higher effect size was found in the unpublished (*d*_+_ = 2.093) than in the published studies (*d*_+_ = 1.127). In the follow-up, the ANOVA did not reach the statistical significance [*Q*_B_(1) = 0.02, *p* = .879; *R*^2^ = 0.0], although with the mean effects showing the same trend than in the posttest (*d*_+_ = 0.755 and 0.829 for the published and unpublished studies, respectively).

**Figure 5:**
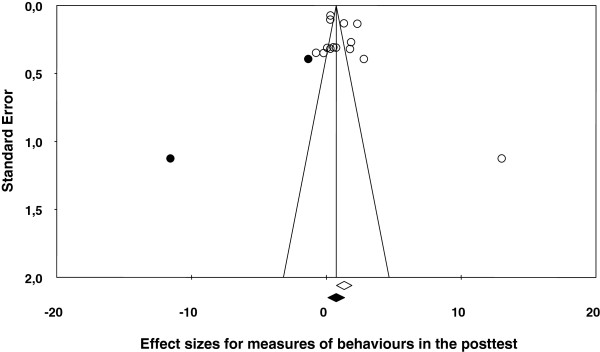
**Funnel plot of effect sizes for measures of behaviours in the posttest.** Full circles are imputed effect sizes from the Duval and Tweedie’s (2000) trim-and-fill method to achieve symmetry in the funnel plot.

The scarce representativeness of the unpublished studies recovered in our meta-analysis led us to examine further whether publication bias might be a threat against our meta-analytic results. With this purpose, a funnel plot was constructed for each one of the four meta-analyses, and the trim-and-fill method [46] was applied, when needed, in order to achieve symmetry in the funnel plot by imputing effect sizes. Figure 5 presents the funnel plot for the effect sizes obtained with measures of behaviours in the posttest. The trim-and-fill method had to impute two new effect sizes (on the left side of the graph) to achieve a symmetric funnel plot. Adding these two adjusted effect sizes led to a decrease of the mean effect, from the original *d*_+_ = 1.33 (see Figure 1) to a *d*_+_ = 0.74 (95% CI: 0.11 and 1.36), that is, a decrease of 44%.

**Figure 6:**
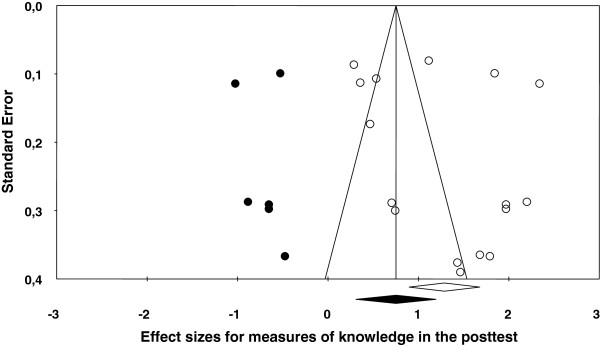
**Funnel plot of effect sizes for measures of knowledge in the posttest.** Full circles are imputed effect sizes from the Duval and Tweedie’s (2000) trim-and-fill method to achieve symmetry in the funnel plot.

**Figure 7:**
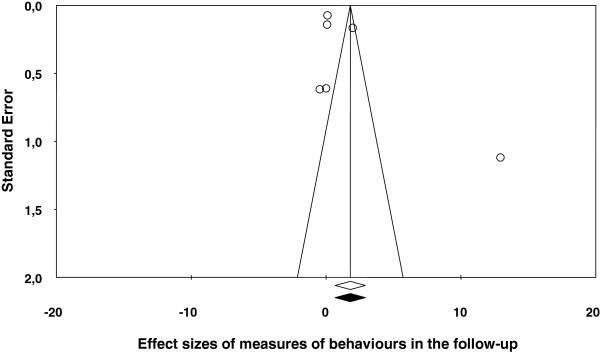
**Funnel plot of effect sizes for measures of behaviours in the follow-up.** The absence of full circles in the graph indicates an approximately symmetric funnel plot.

**Figure 8:**
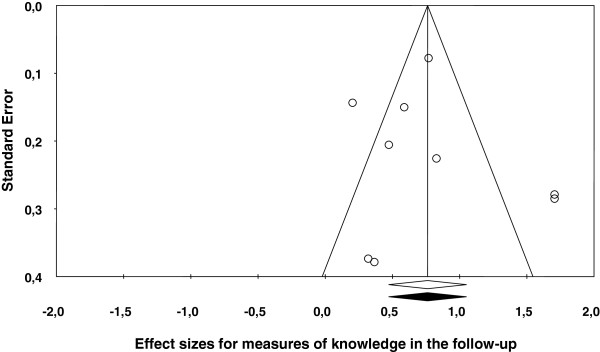
**Funnel plot of effect sizes for measures of knowledge in the follow-up.** The absence of full circles in the graph indicates a symmetric funnel plot.

Figure 6 presents the funnel plot for measures of knowledge in the posttest. The trim-and-fill method had to impute six new effect sizes to achieve symmetry in the funnel plot, giving rise to a decrease in the mean effect from the original *d*_+_ = 1.29 (see Figure 2) to a *d*_+_ = 0.75 (95% CI: 0.31 and 1.19), that is, a decrease of 39.5%. In the follow-up, as shown in Figure 7 and Figure 8 for measures of behaviours and knowledge, respectively, the funnel plot did not depart from symmetry and the trim-and-fill did not have to impute any effect size. Therefore, these analyses point towards the potential existence of publication bias in our meta-analyses in the posttest, but not in those for the follow-up.

### Analyzing moderator variables

In the four meta-analyses, the effect sizes exhibited a large heterogeneity (based on the *Q* statistics and the *I*^2^ indices; see Figures 1, 2, 3, 4), supporting our decision of applying random-effects models. In order to determine which moderator variables were influencing the effect sizes, ANOVAs (for the qualitative variables) and simple meta-regressions (for the quantitative variables) were carried out. The analysis of the moderator variables was applied for the two meta-analyses that included 10 or more studies: behaviours and knowledge in the posttest. In these analyses, the outlying effect size obtained in the Méndez and Gómez’s (2001) study [20] was removed from the statistical analyses for measures of behaviours in the posttest.

### Outcome variable: behaviours in the posttest

Several treatment characteristics were coded in the studies. Tables 3 and 4 present the results of the ANOVAs and meta-regressions, respectively, to examine the influence of moderator variables on the effect sizes. As Table 3 shows, the type of treatment did not reach a statistically significant relationship with the effect sizes (*p* = .525). The majority of the treatments (11 studies) applied postural hygiene (PH) alone, whereas the combination of PH with physiotherapy exercise and PH with physical activity were represented in the analyses with one study only each one. Thus, a so unbalanced distribution of the studies limits the scope of these results. When the studies were classified as a function of the type of postural hygiene, a statistically significant relationship was found with the effect sizes (*p* = .046), with better results exhibited by combining acquisition of knowledge (AK) with posture training habits (PTH; *d*_+_ = 1.466: 95% CI: 0.715 and 2.217) in comparison to AK alone (*d*_+_ = −0.126) and the combination of AK with PTH and stimulation of dynamic postures (*d*_+_ = 0.820). In fact, only the combination of AK with PTH showed a mean effect size statistically significant. Similarly, when analyzing the teaching mode of postural hygiene, statistically significant differences were found (*p* = .010) when comparing theoretical teaching (TT) alone (*d*_+_ = 0.051) with the combination of TT plus practical teaching (*d*_+_ = 1.378). In particular, the combination of TT plus practical teaching showed a statistically significant mean effect, whereas the mean effect for TT alone was practically null. Other qualitative variables related to the treatment characteristics that did not reach a statistically significant relationship with the effect sizes were (see Table 3) the use of external agents (*p* = .393), the use of parents as paraprofessionals (*p* = .688), the use of teachers as paraprofessionals (*p* = .383), and the mode of application of the treatment (*p* = .594).

**Table 3:** Results of the mixed-effects ANOVAs for the qualitative moderator variables on the effect sizes obtained from measures of behaviours in the posttest

**Cluster / Moderator variable**	***k***	***d***_**+**_	**95% C. I.**		**ANOVA results**
			***d***_**l**_	***d***_**u**_	
(A) Treatment characteristics:					
*Type of treatment:*					*Q*_B_(2) = 1.29, *p* = .525
Postural hygiene (PH)	11	0.853	0.256	1.451	*R*^2^ = 0.0
PH + Physiotherapy exercise (PE)	1	1.858	−0.114	3.831	*Q*_W_(10) = 247.72, *p* < .001
PH + Physical activity	1	0.316	−1.594	2.227	
*Type of postural hygiene:*					*Q*_B_(2) = 6.16, *p* = .046
Acquisition of knowledge (AK)	4	−0.126	−1.135	0.884	*R*^2^ = 0.0
AK + Posture training habits (PTH)	7	1.466	0.715	2.217	*Q*_W_(10) = 250.24, *p* < .001
AK + PTH + Stimulat. dynamic postures	2	0.820	−0.541	2.181	
*Teaching method of postural hygiene:*					*Q*_B_(1) = 6.63, *p* = .010
Theoretical teaching (TT)	5	0.051	−0.751	0.853	*R*^2^ = 0.033
TT + Practical teaching	8	1.378	0.764	1.992	*Q*_W_(11) = 259.97, *p* < .001
*External agents:*					*Q*_B_(1) = 0.85, *p* = .393
Yes	7	1.095	0.438	1.752	*R*^2^ = 0.037
No	6	0.636	−0.088	1.360	*Q*_W_(11) = 235.80, *p* < .001
*Parents as paraprofessionals:*					*Q*_B_(1) = 0.16, *p* = .688
Parental involvement	2	0.642	−0.660	1.944	*R*^2^ = 0.0
No parental involvement	11	0.931	0.383	1.478	*Q*_W_(11) = 283.70, *p* < .001
*Teachers as paraprofessionals:*					*Q*_B_(1) = 0.76, *p* = .383
Teacher involvement	4	1.258	0.252	2.265	*R*^2^ = 0.0
No teacher involvement	9	0.719	0.043	1.395	*Q*_W_(11) = 283.97, *p* < .001
*Mode of application:*					*Q*_B_(2) = 1.04, *p* = .594
Indirect intervention	1	0.741	−1.354	2.837	*R*^2^ = 0.0
Direct intervention	9	0.718	0.025	1.412	*Q*_W_(10) = 283.94, *p* < .001
Mixed intervention	3	1.427	0.239	2.616	
(B) Methodological characteristics:					
*Use of pretest measures:*					*Q*_B_(1) = 0.15, *p* = .702
Yes	12	0.915	0.392	1.439	*R*^2^ = 0.0
No	1	0.542	−1.293	2.377	*Q*_W_(11) = 283.51, *p* < .001
*Type of control group:*					*Q*_B_(1) = 0.16, *p* = .688
Active control	2	0.642	−0.660	1.944	*R*^2^ = 0.0
Nonactive control	11	0.931	0.383	1.478	*Q*_W_(11) = 283.70, *p* < .001
*Evaluator blinding:*					*Q*_B_(1) = 7.10, *p* = .008
Blinded evaluator	9	1.310	0.737	1.882	*R*^2^ = 0.062
Not blinded evaluator (or not reported)	4	−0.125	−1.011	0.762	*Q*_W_(11) = 254.47, *p* < .001

*k*: number of studies. *d*_+_: mean effect size. *d*_l_ and *d*_u_: lower and upper confidence limits of the 95% confidence interval around the mean effect size. *Q*_B_: statistic for testing the significance of the moderator variable. *Q*_W_: statistic for testing the model misspecification. *R*^2^: proportion of variance accounted for by the moderator variable. The statistical analyses here presented were carried out excluding the Méndez and Gómez’s (2001) *d* index of 13.033 [20].

**Table 4:** Results of the mixed-effects meta-regressions for the continuous moderator variables on the effect sizes obtained from measures of behaviours in the posttest

**Cluster / Moderator variable**	***k***	**Min.**	**Max.**	**Mean**	***SD***	***b***_**j**_	***Q***_**R**_	***Q***_**E**_**(*df*)**	***R***^**2**^
(A) Treatment characteristics:									
Treatment duration (weeks)	13	1.0	96.0	20.4	33.8	0.001	0.02	283.61(11)***	0.0
Treatment intensity (hours/week)	10	0.2	1.0	0.6	0.4	1.784	3.51^a^	199.91(8)***	0.0
Treatment magnitude (total hours)	10	0.2	6.0	3.2	2.5	0.324	4.87*	192.62(8)***	0.0
(B) Subject characteristics:									
Mean age (years)	11	10.0	12.0	11.0	0.7	−0.662	1.84	251.97(9)***	0.0
Gender (% male)	11	43.3	58.2	50.1	5.2	−0.033	0.29	253.92(9)***	0.0
(C) Methodological characteristics:									
Differential attrition	13	0.0	0.033	0.003	0.009	11.265	0.14	272.07(11)***	0.0
Quality score	13	5.0	6.5	6.1	0.6	1.040	5.87*	259.63(11)***	0.035
(D) Extrinsic characteristic:									
Publication year	13	1984	2011	2000	8.4	0.036	1.20	282.19(11)***	0.0

^a^*p* = .06. * *p* < .05. ** *p* < .01. *** *p* < .001. *k*: number of studies. Min. and Max.: minimum and maximum values of the moderator variable. *SD*: standard deviation of the moderator variable. *b*_j_: unstandardized regression coefficient associated to the moderator variable. *Q*_R_: statistic for testing the significance of the moderator variable. *Q*_E_: statistic for testing the model misspecification. *df*: degrees of freedom. *R*^2^: proportion of variance accounted for by the moderator variable. The statistical analyses here presented were carried out excluding the Méndez and Gómez’s (2001) *d* index of 13.033 [20].

With regards to the continuous variables, the magnitude of the intervention (*p* < .05) showed a positive and statistically significant relationship with the effect sizes, and the intensity of the intervention approached to statistical significance (*p* = .06). Thus, the larger the intensity and the total number of hours of intervention, the better the effectiveness of the treatment. The duration of the treatment did not show a statistical relationship with the effect sizes (see Table 4).

Table 4 also presents the results for two moderator variables related to the participant characteristics in the samples: the mean age and the gender distribution (percentage of males). Anyone of them reached a statistically significant relationship with the effect sizes, although the negative value of their slopes (*b*_j_ = −0.662 and −0.033, respectively) indicated a slight trend to show lower effect sizes as the mean age and the percentage of males increased.

With regards to the methodological characteristics, Table 3 presents the results of applying ANOVAs on several qualitative variables. Statistically significant differences (*p* = .008; *R*^2^ = 0.062) were found when comparing the mean effect obtained by studies that used blinded evaluators (*d*_+_ = 1.310) to those that did not use them (*d*_+_ = −0.125), the first category exhibiting a statistically significant mean effect size. Neither the use of pretest measures (*p* = .702) nor the type of control group (*p* = .688) showed a statistically significant relationship with the effect sizes. However, a trend was found to exhibit lower effect sizes when the treatments were compared to active control groups (*d*_+_ = 0.642) than when using nonactive controls (*d*_+_ = 0.931). Simple meta-regressions were applied on two continuous methodological variables: the differential attrition between the treatment and control groups and the total score obtained in the methodological quality scale (see Table 4). The methodological quality showed a positive, statistically significant relationship with the effect sizes (*p* < .05, *R*^2^ = 0.035), whereas the differential attrition did not show a statistical relationship.

Finally, the publication date did not reach a statistically significant relationship with the effect sizes (see Table 4). A thorough examination of the studies revealed the existence of only a few research teams producing the majority of the studies included in the meta-analysis. The existence of a statistical relationship between research teams and effect sizes can limit the generalizability of the meta-analytic results. With this purpose, an ANOVA was applied once the studies were classified as a function of the research team. As Table 5 shows, a statistically significant relationship was found between research team and effect size (*p* = .002), with the largest mean effect obtained by the Gómez, Méndez et al.’s team (*d*_+_ = 3.145), followed by the Cardon, Geldhof et al.’s team (*d*_+_ = 1.504), and with the other two research teams (USA and South Korea) showing a mean effect statistically not significant (*d*_+_ = −0.463 and 0.205, respectively). When the outlying effect size obtained in the Méndez and Gómez’s (2001) study [20] was removed from the analysis, still a statistically significant relationship was found between research team and effect size (*p* = .035), but in this case only the Belgium team exhibited a mean effect size statistically significant.

**Table 5:** Results of the mixed-effects ANOVA for the qualitative moderator variable “research team” on the effect sizes obtained from measures of behaviours in the posttest

**Research team**	***k***	***d***_**+**_	**95% C. I.**		**ANOVA results**
			***d***_**l**_	***d***_**u**_	
Cardon, Geldhof et al. (Belgium)	7	1.504	0.706	2.302	*Q*_B_(3) = 14.36, *p* = .002
Gómez, Méndez et al. (Spain)	3	3.145	1.786	4.503	*R*^2^ = 0.0
Spence et al. (USA)	2	−0.463	−2.001	1.074	*Q*_W_(10) = 366.01, *p* < .001
Park & Kim (South Korea)	2	0.205	−1.318	1.728	
Excluding the Méndez and Gómez’s (2001) study [20]:					
Cardon, Geldhof et al. (Belgium)	7	1.492	0.828	2.156	*Q*_B_(3) = 8.60, *p* = .035
Gómez, Méndez et al. (Spain)	2	0.642	−0.634	1.917	*R*^2^ = 0.003
Spence et al. (USA)	2	−0.463	−1.759	0.832	*Q*_W_(9) = 249.56, *p* < .001
Park & Kim (South Korea)	2	0.205	−1.074	1.483	

*k*: number of studies. *d*_+_: mean effect size. *d*_l_ and *d*_u_: lower and upper confidence limits of the 95% confidence interval around the mean effect size. *Q*_B_: statistic for testing the significance of the moderator variable. *Q*_W_: statistic for testing the model misspecification. *R*^2^: proportion of variance accounted for by the moderator variable.

### Outcome variable: knowledge in the posttest

Sixteen studies enabled us to obtain an effect size for measures of knowledge in the posttest. The ANOVAs and meta-regressions applied to search for potential moderator variables are shown in Tables 6 and 7, respectively. The majority of the studies applied interventions based on postural hygiene (PH) alone (12 studies; *d*_+_ = 1.301), whereas only three combined PH with physiotherapy exercise (*d*_+_ = 1.521), and one combined PH with physical activity (*d*_+_ = 0.537). The comparison of the three mean effect sizes did not reach a statistical significant result (*p* = .583), although the combination of PH with physiotherapy exercise exhibited the largest mean effect. Neither the type of postural hygiene (*p* = .159), nor the teaching method of postural hygiene (*p* = .669), nor the use of external agents (*p* = .201), nor the use of the parents as paraprofessionals (*p* = .384) reached a statistically significant relationship with the effect sizes. When the studies were classified as a function of whether they used or not teachers as cotherapists, a statistically significant difference was found (*p* = .009; *R*^2^ = 0.497) in favour of the interventions that did not used them (*d*_+_ = 0.730 and 1.544). The mode of application of the treatment influenced the results (*p* = .004; *R*^2^ = 0.481), with the best results for the interventions directly applied by the therapists (*d*_+_ = 1.578), followed by mixed interventions (*d*_+_ = 0.534), that is, interventions where part of the treatment was applied by cotherapists that were family members or teachers who had been trained by the therapist, and part was applied directly by the therapist. The treatment duration showed a negative and statistically significant relationship with the effect sizes (*p* < .01; *R*^2^ = 0.430), suggesting that the lower the number of weeks of treatment, the better the results obtained (see Table 7). However, two studies had very extreme treatment durations (96 weeks both of them) [21,30] in comparison to the remaining studies (range: 1 to 15 weeks). When these two studies were removed from the analysis, the treatment duration did not show a statistically significant relationship with the effect sizes *Q*_R_(1) = 0.77, *p* > .05]. On the other hand, neither the intensity nor the magnitude of the interventions were statistically related to the effect sizes.

**Table 6:** Results of the mixed-effects ANOVAs for the qualitative moderator variables on the effect sizes obtained from measures of knowledge in the posttest

**Cluster / Moderator variable**	***k***	***d***_**+**_	**95% C. I.**		**ANOVA results**
			***d***_**l**_	***d***_**u**_	
(A) Treatment characteristics:					
*Type of treatment:*					*Q*_B_(2) = 1.08, *p* = .583
Postural hygiene (PH)	12	1.301	0.821	1.781	*R*^2^ = 0.0
PH + Physiotherapy exercise (PE)	3	1.521	0.562	2.479	*Q*_W_(13) = 359.20, *p* < .001
PH + Physical activity	1	0.537	−1.060	2.134	
*Type of postural hygiene:*					*Q*_B_(2) = 3.67, *p* = .159
Acquisition of knowledge (AK)	6	1.394	0.810	1.979	*R*^2^ = 0.251
AK + Posture training habits (PTH)	8	1.432	0.954	1.911	*Q*_W_(13) = 210.58, *p* < .001
AK + PTH + Stimulat. dynamic postures	2	0.449	−0.472	1.369	
*Teaching method of postural hygiene:*					*Q*_B_(1) = 0.18, *p* = .669
Theoretical teaching (TT)	6	1.407	0.736	2.077	*R*^2^ = 0.0
TT + Practical teaching	10	1.225	0.730	1.720	*Q*_W_(14) = 341.28, *p* < .001
*External agents:*					*Q*_B_(1) = 1.63, *p* = .201
Yes	10	1.119	0.694	1.545	*R*^2^ = 0.244
No	6	1.592	1.005	2.178	*Q*_W_(14) = 268.85, *p* < .001
*Parents as paraprofessionals:*					*Q*_B_(1) = 0.76, *p* = .384
Parental involvement	1	1.972	0.385	3.559	*R*^2^ = 0.003
No parental involvement	15	1.244	0.842	1.647	*Q*_W_(14) = 377.45, *p* < .001
*Teachers as paraprofessionals:*					*Q*_B_(1) = 6.77, *p* = .009
Teacher involvement	5	0.730	0.231	1.230	*R*^2^ = 0.497
No teacher involvement	11	1.544	1.189	1.898	*Q*_W_(14) = 173.17, *p* < .001
*Mode of application:*					*Q*_B_(2) = 11.15, *p* = .004
Indirect intervention	1	0.289	−0.793	1.371	*R*^2^ = 0.481
Direct intervention	12	1.578	1.234	1.923	*Q*_W_(13) = 144.57, *p* < .001
Mixed intervention	3	0.534	−0.118	1.185	
(B) Methodological characteristics:					
*Type of control group:*					*Q*_B_(1) = 1.65, *p* = .198
Active control	2	1.972	0.860	3.083	*R*^2^ = 0.028
Nonactive control	14	1.194	0.783	1.605	*Q*_W_(14) = 367.82, *p* < .001
*Evaluator blinding:*					*Q*_B_(1) = 0.35, *p* = .553
Blinded evaluator	10	1.372	0.902	1.841	*R*^2^ = 0.078
Not blinded evaluator (or not reported)	6	1.133	0.503	1.764	*Q*_W_(14) = 305.94, *p* < .001

*k*: number of studies. *d*_+_: mean effect size. *d*_l_ and *d*_u_: lower and upper confidence limits of the 95% confidence interval around the mean effect size. *Q*_B_: statistic for testing the significance of the moderator variable. *Q*_W_: statistic for testing the model misspecification. *R*^2^: proportion of variance accounted for by the moderator variable.

**Table 7:** Results of the mixed-effects meta-regressions for the continuous moderator variables on the effect sizes obtained from measures of knowledge in the posttest

**Cluster / Moderator variable**	***k***	**Min.**	**Max.**	**Mean**	***SD***	***b***_**j**_	***Q***_**R**_	***Q***_**E**_**(*df*)**	***R***^**2**^
(A) Treatment characteristics:									
Treatment duration (weeks)	15	1.0	96.0	17.5	32.1	−0.012	6.60**	144.46(13)***	0.430
Treatment intensity (hours/week)	12	0.2	2.4	0.9	0.6	−0.059	0.03	142.28(10)***	0.0
Treatment magnitude (total hours)	12	0.2	19.0	5.0	5.0	−0.001	0.00	128.72(10)***	0.026
(B) Subject characteristics:									
Mean age (years)	14	8.0	12.0	1.2	1.2	0.130	0.48	345.18(12)***	0.0
Gender (% male)	14	45.8	58.2	3.7	3.7	0.029	0.20	384.63(12)***	0.0
(C) Methodological characteristics:									
Differential attrition	16	0.0	0.033	0.004	0.009	−24.205	1.14	355.39(14)***	0.0
Quality score	16	3.4	7.5	6.0	1.1	0.282	2.71	267.05(14)***	0.177
(D) Extrinsic characteristic:									
Publication year	16	1984	2011	2003	8.4	−0.014	0.28	370.92(14)***	0.0

* *p* < .05. ** *p* < .01. *** *p* < .001. *k*: number of studies. Min. and Max.: minimum and maximum values of the moderator variable. *SD*: standard deviation of the moderator variable. *b*_j_: unstandardized regression coefficient associated to the moderator variable. *Q*_R_: statistic for testing the significance of the moderator variable. *Q*_E_: statistic for testing the model misspecification. *df*: degrees of freedom. *R*^2^: proportion of variance accounted for by the moderator variable.

With regards to the participant characteristics, simple meta-regressions applied on the mean age (in years) of the samples and on the percentage of males did not show a statistically significant relationship with the effect sizes (see Table 7). Similar results were found when the influence of methodological variables was tested: neither the type of control group (*p* = .198), nor the use of blinded evaluators (*p* = .553), nor the differential attrition, nor the quality score showed a statistically significant relationship with the effect sizes (see Tables 6 and 7).

Finally, two extrinsic variables were analyzed: the publication year and the research team. As Table 7 shows, the publication year did not show a statistically significant relationship with the effect sizes. With regards to the research team, a highly statistically significant result was obtained (*p* < .001) with a large proportion of variance accounted for (*R*^2^ = 0.718). The Gómez, Méndez et al.’s team exhibited the largest mean effect (*d*_+_ = 2.043) and the Kovacs et al.’s team the lowest one (*d*_+_ = 0.289) (see Table 8).

**Table 8:** Results of the mixed-effects ANOVA for the qualitative moderator variable “research team” on the effect sizes obtained from measures of knowledge in the posttest

**Research team**	***k***	***d***_**+**_	**95% C. I.**		**ANOVA results**
			***d***_**l**_	***d***_**u**_	
Cardon, Geldhof et al. (Belgium)	6	0.901	0.541	1.261	*Q*_B_(5) = 26.49, *p* < .001
Gómez, Méndez et al. (Spain)	4	2.043	1.600	2.485	*R*^2^ = 0.718
Spence et al. (USA)	2	1.450	0.681	2.219	*Q*_W_(10) = 71.28, *p* < .001
Vidal et al. (Spain)	1	0.469	−0.388	1.327	
Park & Kim (South Korea)	2	1.739	0.985	2.492	
Kovacs et al. (Spain)	1	0.289	−0.516	1.094	

*k*: number of studies. *d*_+_: mean effect size. *d*_l_ and *d*_u_: lower and upper confidence limits of the 95% confidence interval around the mean effect size. *Q*_B_: statistic for testing the significance of the moderator variable. *Q*_W_: statistic for testing the model misspecification. *R*^2^: proportion of variance accounted for by the moderator variable.

## Discussion

The main objective of our study was to determine the effectiveness of preventive physiotherapy treatments for back care in children and adolescents, as well as to examine the treatment, subject, context, methodological and extrinsic characteristics that may be moderating the results. A total of 23 studies met our selection criteria and standardized mean differences were calculated from each of them by comparing a treatment to a control group. Separate meta-analyses were carried out for effect sizes obtained from measures of behaviours and knowledge, both in the posttest and in the follow-up. Moderator analyses were carried out for behaviours and knowledge in the posttest.

### Relating to behaviours in the posttest

Although the mean effect size obtained for measures of behaviours in the posttest was *d*_+_ = 1.33, when an outlying effect size was removed from the analysis [20], the mean effect decreased to *d*_+_ = 0.89, although still being highly statistically significant. The evidence of an asymmetric funnel plot led to suspect that publication bias might be a threat for the meta-analytic results. So, the trim-and-fill method gave a more conservative estimate of the true effect of preventive interventions for LBP, with a mean effect of *d*_+_ = 0.74. From the results obtained in the analysis of the treatment modalities used, the type of postural hygiene seems to be a relevant moderator of effect size, with the combination of knowledge acquisition plus posture training habits being the most efficacious. The teaching method of postural hygiene also influenced the effect sizes, with better results when theoretical and practical teachings were combined. The hypothesis that the duration, intensity and magnitude of the treatment influence the results has been partially confirmed by our results, enabling us to conclude that, the higher the intensity and magnitude, the more efficacious the treatment. Previous research have shown that the interventions improve their benefits when they include the figures of parents or teachers as cotherapists [20,27]. However, our hypotheses on the positive influence of using external agents and parents and teachers as paraprofessionals were not supported by our results. Regarding the participant characteristics in the samples, the hypothesis that the age of subjects would negatively influence the results was not supported. In the same vein, the gender distribution of the sample did not influence the results, not supporting our hypothesis. Our hypothesis that studies with a nonactive control group would have higher effect sizes than those with an active control group was not confirmed, although the results pointed in that direction. This result must be interpreted very cautiously because only a few studies used an active control group [20,23,40].

### Relating to knowledge in the posttest

For knowledge acquisition a positive and highly statistically significant mean effect size was obtained, *d*_+_ = 1.29, although the trim-and-fill method had to impute six new effect sizes to achieve symmetry in the funnel plot. Therefore, a more conservative estimate of the true effect that controls for publication bias is *d*_+_ = 0.75, still statistically significant and of a large magnitude. The different treatment modalities did not seem to affect the effect sizes. The hypothesis that the duration, intensity and magnitude of the treatment may influence the results was not supported by our results. Using external agents and the presence of family paraprofessionals did not influence the effect size magnitude. However the presence of teachers as cotherapists did affect the results, but inversely to our expectations. The mode of application influenced the effect size magnitude, with direct interventions obtaining the best results. Regarding the participant characteristics, neither the gender nor the age of the subjects influenced the results. In the case of gender, Rebolho [58] has shown a higher level of knowledge acquisition for females than for males. With regards to the type of control group, this variable did not influence the results, and therefore we could not confirm our hypothesis of a lower effect size for studies that compare the intervention with an active control group than when the control group is nonactive.

### Results in the follow-up

The maintenance of the changes due to the interventions was assessed in a few studies that enabled us to calculate effect sizes in the follow-up. With regards to measures of behaviours, the mean effect size was positive and of a large magnitude, *d*_+_ = 1.80. However, when the extremely outlying effect size obtained in the Méndez and Gómez’s (2001) [20] study was removed from the analysis, the mean effect decreased to *d*_+_ = 0.44 and did not reach the statistical significance. In the case of measures of knowledge, the mean effect size was of large magnitude and statistically significant, *d*_+_ = 0.76. Therefore, with regards to behaviour measures we cannot be sure that the benefits of the interventions will be maintained over time.

### Limitations of the meta-analysis

It is important to note some limitations of our meta-analysis. The absence of a more detailed description in the primary studies about such important characteristics as the treatment techniques, its mode of application, caused us uncertainty in our coding process. On the other hand, the small number of studies in our meta-analysis makes our results be interpreted with caution and be taken as provisional, pending the publication of new studies in this field. In addition, the limited number of studies has prevented to formulate an explanatory model of the effect sizes variability, by applying a multiple meta-regression model. Another limitation is the evidence of publication bias in some of our meta-analytic results, inviting us to a cautious interpretation of the results and to take the effect estimates obtained with the trim-and-fill method as more appropriate. Finally, a circumstance that limits the generalizability of our results is the scarce number of research teams that have carried out the studies included in our meta-analysis.

### Implications for clinical practice

The main implication of our results for clinical practice is that preventive physiotherapy interventions for back care should combine knowledge and training of postural habits with physiotherapy exercises.

### Implications for future research

The results of our meta-analysis allow us to propose some recommendations for future research in this field. Firstly, it is advisable that future studies report more information regarding the characteristics of the treatments applied. Furthermore, with the purpose of obtaining important data relating to the maintenance of the changes, researchers should conduct follow-ups. One of the 23 studies [20] exhibited a very large effect size in measures of behaviours, both in the posttest (*d* = 13.033) and in the follow-up (*d* = 12.957). This study was who exhibited the longest intensity (2.4 hours per week), the largest magnitude (a total of 19 hours of intervention), the only one that included homework and, in addition, it used family and teachers as cotherapists. In addition, this study achieved the maximum quality score out of all of the studies of the meta-analysis. Although this study is not representative of the set of studies included in the meta-analysis, the extremely large effectiveness found in it advises that new studies try to replicate their results by implementing interventions similar to that applied in this study.

## Conclusion

In conclusion, the combined treatment of postural hygiene with physiotherapy exercise has been proven to be the most efficacious in relation to the two outcome variables: behaviours and knowledge. The treatments were successful in significantly increasing the behaviours and knowledge acquired both in the posttest and in follow-up.

## Competing interests

The authors declare that they have no competing interests.

## Authors’ contributions

All authors contributed to conception and design, acquisition, analysis and interpretation of data and drafting of the manuscript. AGC and JSM participated in the critical revision of the manuscript for important intellectual content. ICM and JSM performed statistical analyses. All authors read and approved the final manuscript.

## Pre-publication history

The pre-publication history for this paper can be accessed here:

http://www.biomedcentral.com/1471-2474/13/152/prepub

## Supplementary Material

Additional file 1 Results of the search from some of the databases consulted. Click here for file

Additional file 2 **Flow chart of the selection of studies for the meta-analysis.** PPT: preventive physiotherapy treatments Click here for file

Additional file 3 PRISMA Checklist. Click here for file

Additional file 4 Methodological quality of the 19 papers. Click here for file
