# Supplementary material for: Preventive physiotherapy interventions for back care in children and adolescents: a meta-analysis
Source: BMC Musculoskelet Disord. 2012 Aug 21;13:152. doi: 10.1186/1471-2474-13-152 (PMC3488493; doi:10.1186/1471-2474-13-152)
Supplement: Additional file 1 — Results of the search from some of the databases consulted. [file 1471-2474-13-152-S1.doc]

|  | | |
| --- | --- | --- |
| **Additional file 1**  Results of the search from some of the databases consulted | | |
| **Databases** | **Key words** | **Results** |
| Cochrane Library | PHASE 1: (1) childhood; (2) adolescence; (3) children; (4) #1 OR #2 OR #3.  PHASE 2: (1) treatment; (2) prevention; (3) education; (4) “postural hygiene program”; (5) “physical education”; (6) “back education” ; (7) “Posture education”; (8) “Back function”; (9) physiotherapy; (10) ergonomics; (11) ”physical therapy”; (12) “exercise therapy” ; (13) “back care”; (14): #1 OR #2 OR #3 OR #4 OR #5 OR #6 OR #7 OR #8 OR #9 OR #10 OR #11 OR #12 OR #13.  PHASE 3: (1) “back pain”; (12) “low back pain” #1 OR #2.  TOTAL PHASES: 1 AND 2 AND 3. | 152 |
| Medline | PHASE 1: (1) Child*; (2) Adolescen*; (3) #1 OR #2  PHASE 2: (1) treatment; (2) prevention; (3) education; (4) “postural hygiene program”; (5) “physical education”; (6) “back education” ; (7) “Posture education”; (8) “Back function” ; (9) physiotherapy; (10) ergonomics; (11) ”physical therapy”; (12) “exercise therapy” (13) promotion; (14) behaviour; (15) medical attention; (16) posture; (17): #1 OR #2 OR #3 OR #4 OR #5 OR #6 OR #7 OR #8 OR #9 OR #10 OR #11 OR #12 OR #13 OR #14 OR #15 OR #16 OR #17.  PHASE 3: (1) “back pain”; (2)”low back pain”; (3) “back care”.  TOTAL PHASES: 1 AND 2 AND 3 | 259 |
| Web  of Science | PHASE 1: (1) Child*; (2) Adolescen*; (3) #1 OR #2  PHASE 2: (1) treatment; (2) prevention; (3) education; (4) “postural hygiene program” (5) “physical education”; (6) “back education” ; (7) “Posture education”; (8) “Back function” ; (9) physiotherapy; (10) ergonomics; (11) ”physical therapy”; (12) “exercise therapy” (13) promotion; (14) behaviour; (15) medical attention; (16) posture; (17): #1 OR #2 OR #3 OR #4 OR #5 OR #6 OR #7 OR #8 OR #9 OR #10 OR #11 OR #12 OR #13 OR #14 OR #15 OR #16 OR #17.  PHASE 3: (1) “back pain”; (2)”low back pain”; (3) “back care”.  TOTAL PHASES: 1 AND 2 AND 3 | 431 |
