# Supplementary material for: Preventive physiotherapy interventions for back care in children and adolescents: a meta-analysis
Source: BMC Musculoskelet Disord. 2012 Aug 21;13:152. doi: 10.1186/1471-2474-13-152 (PMC3488493; doi:10.1186/1471-2474-13-152)
Supplement: Additional file 2 — Flow chart of the selection of studies for the meta-analysis. PPT: preventive physiotherapy treatments [file 1471-2474-13-152-S2.doc]

| **Additional file 2** |
| --- |
| Flow chart of the selection of studies for the meta-analysis. PPT: preventive physiotherapy treatments |

Records identified by searching databases Cochrane Library (152), Medline (259), Web of Science (431), PEDro (9), IME (105)

(n = 956)

Additional records identified via other sources

(n = 11)

Records screened

(n = 967)

Records after removal of duplicates

(n = 967)

Full-text articles

assessed for eligibility

(n=62)

Full-text articles excluded

(n=43)

> 18 years (n=16)

No PPT (n=9)

Absence statistical data (n=8)

All subjects with pain (n=4)

All subjects with deformity (n=2)

No control group (n=4)

Articles included in the quantitative

synthesis (meta-analysis)

(n=19)

Studies included in the quantitative synthesis (meta-analysis)

(n=23)

Identification

Records excluded

(n = 905)

Screening

Eligibility

Included
