# Supplementary material for: Preventive physiotherapy interventions for back care in children and adolescents: a meta-analysis
Source: BMC Musculoskelet Disord. 2012 Aug 21;13:152. doi: 10.1186/1471-2474-13-152 (PMC3488493; doi:10.1186/1471-2474-13-152)
Supplement: Additional file 4 — Methodological quality of the 19 papers. [file 1471-2474-13-152-S4.doc]

| **Additional file 4**  Methodological quality of the 19 papers | | | | | | | | | |
| --- | --- | --- | --- | --- | --- | --- | --- | --- | --- |
| **Article (19)** | **1** | **2** | **3** | **4** | **5** | **6** | **7** | **8** | **Total** |
| Cardon et al,^19^ (2000) | 0.5 | 0 | 1 | 1 | 1 | 1 | 1 | 1 | 6.5 |
| Cardon et al,^27^ (2001) | 0.5 | 0 | 1 | 1 | 1 | 1 | 1 | 1 | 6.5 |
| Cardon et al,^28^ (2002a) | 0.5 | 0 | 1 | 1 | 1 | 1 | 1 | 1 | 6.5 |
| Cardon et al,^29^ (2002b) | 0.5 | 0 | 1 | 0.995 | 1 | 1 | 1 | 1 | 6.495 |
| Cardon et al,^30^ (2007) | 0.5 | 0 | 1 | 0.927 | 1 | 1 | 1 | 1 | 6.427 |
| Cardoso^33^ (2009) | 0.5 | 0 | 1 | 1 | 1 | 1 | 1 | 1 | 6.5 |
| Dolphens et al,^52^ (2011) | 0.5 | 0 | 1 | 0.99 | 1 | 1 | 1 | 1 | 6.49 |
| Geldhof et al,^21^ (2006) | 0,5 | 0 | 1 | 0.902 | 1 | 1 | 1 | 1 | 6.402 |
| Geldhof et al,^53^ (2007a) | 0.5 | 0 | 1 | 0.932 | 1 | 1 | 1 | 1 | 6.432 |
| Geldhof et al,^54^ (2007b) | 0.5 | 0 | 1 | 0.902 | 1 | 1 | 1 | 1 | 6.402 |
| Geldhof et al,^55^ (2007c) | 0.5 | 0 | 1 | 0.902 | 1 | 1 | 1 | 1 | 6.402 |
| Gómez and Méndez^23^ (2000a) | 0.5 | 1 | 1 | 1 | 1 | 1 | 1 | 1 | 7.5 |
| Gómez and Méndez^40^ (2000b) | 0.5 | 1 | 1 | 1 | 1 | 1 | 1 | 0 | 6.5 |
| Kovacs et al,^56^ (2011) | 0.5 | 0 | 1 | 0.940 | 0 | 0 | 1 | 0 | 3.44 |
| Martínez^34^ (2007) | 0.5 | 0 | 1 | 1 | 1 | 1 | 1 | 1 | 6.5 |
| Méndez and Gómez^20^ (2001) | 0.5 | 1 | 1 | 1 | 1 | 1 | 1 | 1 | 7.5 |
| Park and Kim^41^ (2011) | 0 | 0 | 1 | 1 | 1 | 0 | 1 | 1 | 5 |
| Spence et al,^22^ (1984) | 0.5 | 0 | 1 | 1 | 1 | 0 | 1 | 0 | 5.5 |
| Vidal et al,^24^ (2009) | 0.5 | 0 | 1 | 1 | 1 | 0 | 1 | 0 | 4.5 |
| **1: Randomized;** 1 = the subjects were randomly assigned to the experimental conditions; 0.5 = the groups (not the subjects) were randomly assigned to the experimental conditions; 0 = neither the subjects nor the groups were randomly assigned to the experimental conditions. **2: type of control group;** 1 **=** active control; 0 **=** inactive control. **3: Total sample size;** 1 = *N* ≥ 30; 0.5 = 16 ≤ *N* < 29; 0 = *N* < 16. **4: Experimental attrition;** this is computed as 1 – treatment group attrition. **5: Intention-to-treat analysis;** 1: yes; 0: no. **6: Evaluator blinding;** 1: yes; 0: no. **7: homogenous assessment;** 1: yes; 0: no. **8: Inter-rater reliability;** 1: yes; 0: no. | | | | | | | | | |
